# From dependence to self-reliance: The future of the global tuberculosis response

**DOI:** 10.1371/journal.pmed.1004824

**Published:** 2025-11-21

**Authors:** Petra Heitkamp, Obioma Chijioke-Akaniro, Madhukar Pai

**Affiliations:** 1 TB Public-Private Mix Learning Network, Research Institute of the McGill University Health Centre, Montreal, Québec, Canada; 2 National Tuberculosis, Leprosy and Buruli Ulcer Control Programme, Federal Ministry of Health, Abuja, Nigeria; 3 School of Population and Global Health, McGill University, Montreal, Québec, Canada; 4 McGill International Tuberculosis Center, Montreal, Québec, Canada

## Abstract

In this Perspective, Madhukar Pai and colleagues highlight that while international health aid cuts are impacting the tuberculosis response, they also present an opportunity to shift towards a more equitable, resilient, and self-reliant TB response, led by high-burden countries.

## Teaser

Just as tuberculosis services were recovering after the COVID-19 pandemic disruptions, abrupt funding cuts by G7 nations are putting progress at risk. These trends, while perilous, also reveal a turning point toward a more equitable, resilient, and self-reliant TB response, led by high-burden countries.

## Introduction

Before the COVID-19 pandemic, tuberculosis (TB) was the world’s leading infectious killer, causing 1.34 million deaths in 2019. During the pandemic, COVID-19 took the top spot, while simultaneously disrupting routine TB services [1]. While TB programs have since begun to recover, abrupt funding cuts in 2025 threaten to undo years of progress.

The G7 nations plan to reduce aid spending by 28% in 2026 compared with 2024, the biggest cut in aid since the group’s creation in 1975 [2]. Donor funding for TB in low- and middle-income countries has remained largely static since 2013, at about US$1.1 billion per year. Although domestic financing accounts for 25%–94% of TB program costs (depending on the country), many critical activities, such as community outreach, specimen transportation, and TB preventive treatment, rely on donor support. Since donor contributions make up a greater share of TB financing than of overall health spending, TB programs remain acutely exposed to donor volatility [3]. Funding cuts are also impacting the global AIDS response [4], and any scale-back of HIV services will consequently impact TB as well, given that HIV is a major risk factor for TB. As of 22 November 2025, only about 11.3bn have been pledged by donors towards the Global Fund’s targeted budget of $18bn for the 2026–2028 replenishment.

## Real-world impacts of aid cuts on TB programs

The abrupt withdrawal of USAID funding in early 2025 has caused the deepest shock to global health, including TB programs, with the United Kingdom, Switzerland, and others also scaling back support. Unlike gradual phase-outs that allow governments and partners to adjust, this sudden suspension of US support has left programs scrambling to fill critical gaps [5]. Early community surveys [6] document immediate program shutdowns, supply shortages, and reduced access to care.

Recent modeling studies illustrate the scale of the damage. As of October 8, 2025, the TB Impact Counter estimated around 47,900 excess TB deaths and 60,800 additional cases relative to projected pre-suspension trends (reference start date of January 24, 2025) [7]. One modeling study covering 79 countries projected that ending USAID support could cause 1.4 million excess TB episodes and ~537,700 excess deaths by 2035 [8]. Taking into consideration the potential impact of funding cuts to 26 high-burden TB countries, others have estimated that termination of US funding could result in an estimated 10.6 million additional TB cases and 2.2 million additional TB deaths during the period 2025–2030 [5].

These projections are already being felt on the ground, where lifesaving TB services for marginalized populations are shrinking, advocacy capacity has eroded, and ability to scale innovations has reduced. International institutions have acknowledged the crisis and urged governments to safeguard TB financing [9]. The Global Fund has provided policy guidance to mitigate disruptions, introducing efficiency and prioritization measures in grants, procurement, and supply chain management [10]. Yet, while efficiency gains can stretch limited resources, they cannot compensate for large-scale donor withdrawals. Even in countries where publicly funded TB services have remained intact, community-based initiatives have been disproportionately affected.

The funding shock has also impacted research and innovation, with many global health-oriented National Institutes of Health grants terminated or paused [11]. In South Africa, Nigeria, and other high-burden settings, US funding cuts and stop-work orders have forced laboratories to suspend studies, halt field trials, and redirect scarce resources to basic service delivery. Just as the TB community was gaining momentum to scale up new diagnostics, shorter drug regimens, and vaccine trials, it has been pushed back into a survival phase.

## A moment of disruption: Risks and opportunities

These disruptions, while perilous, also reveal a turning point for building a more equitable, resilient, and self-reliant TB response, led by countries that bear the highest burden. As traditional donors scale back, funding channels and priorities are being reshaped by new political and economic realities. There are new risks and new opportunities.

Among those that introduce new risks, we need to consider differential impacts on emerging versus less developed economies. Countries such as Brazil, Russia, India, China, and South Africa are less reliant on aid and can withstand the current funding cuts better than less wealthy nations. Also, these middle-income nations are aligning health investments with economic and strategic interests. Funders and governments are more likely to support large, visible projects that may create jobs and generate revenues (e.g., manufacturing hubs, digital systems, artificial intelligence). TB elimination, however, hinges on social protection, community outreach, food security, and support for vulnerable populations, areas often seen as nonrevenue generating. Sub-Saharan Africa, with the world’s highest TB/HIV coinfection rates, is at risk of being underfunded in this transition. Consequently, increased TB transmission remains a serious public health threat, intensified by internal and cross-border migration from high-incidence areas.

In terms of opportunities, there is great potential for high TB burden countries to lead the global TB response. Countries with high TB burden, such as China, India, and Indonesia, are already showing that progress to ambitious End TB targets (which aim to reduce TB incidence by 80%, TB deaths by 90%, and to eliminate catastrophic costs for TB-affected households by 2030) is possible with strong political leadership, innovative financing, and increased domestic funding through taxation, insurance schemes, and public–private partnerships. This was recently reinforced by Health Ministers of four of the largest high TB burden countries, who wrote “To end TB, time for us to own our disease response and financing for health” [12].

Another opportunity is the emergence of global South innovations and manufacturing. While international funding is shrinking, high TB burden countries will need more affordable solutions. Investments by the Global South countries are starting to pay off. Affordable, locally produced solutions have emerged, including near point-of-care technologies (e.g., MiniDock (Pluslife), TrueNAT (Molbio), and UniAmp (Huwel Lifesciences)) from China and India, digital x-ray and computer-aided detection tools (e.g., Qure AI, DeepTek), and improved infection testing (e.g., SIILTBICY from India), alongside a growing range of generic, affordable, shorter TB regimens. These examples illustrate that the future of TB innovation is increasingly being shaped in the Global South, by countries bearing the greatest disease burden.

Yet another opportunity lies in the integration of TB services within primary healthcare [13]. As funding for vertical programs becomes scarce, countries will be forced to do more with less funding, using routine healthcare infrastructure. Integration of TB and HIV care within routine primary health services could make TB services more sustainable and less donor-dependent in the long term.

Lastly, TB must be reframed as an investment, not aid. TB research and innovation generate global public goods that ensure benefits across all health systems. Investing in TB elimination is not charity; it is a cornerstone of global health security. Preventing TB by investing in people and the workforce builds social capital and drives economic productivity. Every US$1 invested in TB yields US$46 in benefits [14]. Positioning TB as an economic and social investment can help mobilize finance ministries, not just health ministries, around a shared growth agenda. For the Global South, embedding TB within economic and broader development priorities—housing, nutrition and food security, universal health coverage, and primary care agendas—can yield sustainable returns and will be appealing to multilateral banks and regional financing mechanisms.

## Conclusions

Abrupt aid cuts have exposed how fragile and donor-dependent the fight against TB remains. No disease affecting the poorest should be so vulnerable to political decisions beyond its borders. As global health financing evolves, the TB response must redefine itself as a strategic investment in health security, equity, and universal health coverage, while leveraging progress in new innovations. The future of the End TB response will not be decided in Washington or Geneva, but in New Delhi, Abuja, Pretoria, and Jakarta, where political will, domestic investments, and innovation can make sure that a TB-free world will be realized, not because of the benevolence from the Global North but more because of strategic self-determination and leadership from the Global South.
